# Establishing Compliance between Spectral, Colourimetric and Photometric Indicators in Resazurin Reduction Test

**DOI:** 10.3390/bioengineering10080962

**Published:** 2023-08-14

**Authors:** Alexander V. Sychev, Anastasia I. Lavrova, Marine Z. Dogonadze, Eugene B. Postnikov

**Affiliations:** 1 Research Center for Condensed Matter Physics, Kursk State University, Radishcheva St. 33, 305000 Kursk, Russia; 2 Saint-Petersburg State Research Institute of Phthisiopulmonology, Lygovsky av. 2-4, 191036 Saint-Petersburg, Russia; 3 Faculty of Medicine, Saint-Petersburg State University, Universitetskaya emb 7-9, 199034 Saint-Petersburg, Russia; 4 Department of Theoretical Physics, Kursk State University, Radishcheva St. 33, 305000 Kursk, Russia

**Keywords:** REMA, resazurin, resorufin, microbiological analysis, minimal inhibitory concentration

## Abstract

The resazurin reduction test is one of the basic tests for bacterial culture viability and drug resistance endorsed by the World Health Organisation. At the same time, conventional spectrophotometric and spectrofluorimetric methods demand rather bulky and expensive equipment. This induces a challenge for developing simpler approaches to sensor systems that are portable and applicable in resource-limited settings. In this work, we address two such alternative approaches, based on the colour processing of the microbiological plate’s photographic images and single-channel photometry with a recently developed portable microbiological analyser. The key results consist of establishing a sequential linear correspondence between the concentration of resorufin produced due to the reduction of resazurin by viable bacteria as determined by the UV-Vis studies, the intensity of the a* channel of the CIE L*a*b* colour space and the transmitted light intensity registered by a luxmeter under the LED illumination with a yellow colour filter. This route is illustrated with the chemical system “Hydrazine hydrate – resazurin”, isolating the target colour change-inducing reaction and the test of determining the minimal inhibition concentration of the antibacterial first-line drug isoniazid acting on the culture of the H37Rv strain of *M. tuberculosis*.

## 1. Introduction

The resazurin reduction test, first proposed by Pesch and Simmert [1] for testing milk quality, respectively, in terms of bacterial contamination and further as a rapid antibiotic sensitivity test [2,3,4], has recently become one of the basic methods for the determination of the viability of microorganisms applicable to monitor cell viability and metabolic functions [5,6] and proliferation assays [7]. Its principle is based on the conversion of the blue indicator dye resazurin (7-hydroxy-3h-phenoxazin-3-one 10-oxide, also known as Alamar Blue) to resorufin (7-hydroxy-3h-phenoxazin-3-one), having a pink colour (the latter can also be reduced further to colourless dihydroresorufin, 7-hydroxy-1,2-dihydro-3H-phenoxazin-3-one). This reaction is maintained in biological applications by the activity of intracellular species (such as NAD(P)H) in the presence of mitochondrial and cytoplasmic reductases of living cells. Due to its simplicity and inexpensiveness, the quantitative colourimetric test is still used not only in the dairy industry [8] but also in biomedical research for screening antibacterial compounds, e.g., in application to drugs acting on *M. tuberculosis* [9,10] and diarrheagenic bacterial strains [11], revealing wide-spectrum drug resistivity [12].

The respective process can be characterised also quantitatively using either UV-Vis spectroscopy or fluorometry. The first approach addresses the difference between the absorption maxima of resazurin (within the range 596–604 nm) and resorufin (about 570nm), while the second one refers to the fact that resorufin is a highly fluorescent substance (the excitation and emission peaks are at 571nm and 584nm, respectively) in contrast to non-fluorescent resazurin. Among the most demanded modern applications of such test systems, one can list antibiotic susceptibility testing and resistant bacteria detection [13], especially for studying the drug resistance of *Mycobacterium tuberculosis* [14,15,16,17] (so-called resazurin microtiter assay, REMA). Such systems are also widely used in new drug development aimed at overcoming problems urgently for public health, ecological applications of hydrological environment monitoring (e.g., water–sediment interactions and microbial metabolic activity [18,19], characterising sample storage [20]), experiments in radiobiology [21], the cytotoxic and cytostatic exploration of human cells [22,23], etc.

Nevertheless, the feature of this indicator’s colour change does not remain among purely qualitative methods of screening-type studies but experiences the dawn as a quantitative method [24,25,26] that is in line with the recently growing interest in colourimetry in analytical chemistry [27]. This interest is supported by the variety of advantages that colourimetry has in comparison with purely spectral measurements. Some of them originate from the technical restrictions of the range of applicability of UV-Vis and fluorescence-based methods: (i) It is known that resorufin’s fluorescence nonlinearly depends on the concentration of resazurin and can be even quenched by its high concentrations [28] and, moreover, as it was argued recently [29], the registered signal’s intensity can depend on the presence of auxiliary compounds, is strictly instrument-dependent, i.e., specific for a particularly used apparatus, and cannot be calibrated relative to a blank sample signal. (ii) The UV-Vis technique is free from the latter limitations, but absorption spectra are also affected by high concentrations of resazurin and the properties of a solute medium [30,31,32]. (iii) There are technical and financial reasons that demand a search for cheaper and more mobile implementations [13]. In particular, standard spectrophotometrical microplate readers are rather expensive and bulky. This prevents their wide usage in field studies, say, for environment monitoring, in the countryside and in low-income countries [13]. For example, the demand for developing cheap and easy-to-perform kits for *Mycobacterium tuberculosis* drug-resistance testing [33] is an urgent problem namely for such regions.

To overcome the complications mentioned above, the development of a purely colourimetric image-based approach was demanded. Among the first approaches, the processing of images obtained with a digital scanner [34] can be mentioned; however, certain complications were claimed for the usage of the resazurin-based test due to a complex colour response in the RGB colour space in comparison with spectrophotometric data. Despite developing several variants of combined numerical processing multichannel data [35,36], there is an understanding that other colour spaces better adjusted to the absorption spectral properties of resazurin and resorufin could be preferred. Among such alternatives, the developed by the International Commission on Illumination (abbreviated as CIE for its French name, Commission Internationale de l’Éclairage), the tristimuli-based CIE L*a*b* colour space [37] attracts special attention [24,32]. It is a device-independent, “standard observer” model, which maps three distributions of wavelength ranges in the electromagnetic visible spectrum into the variables of lightness (L∗), green–magenta (a∗) and blue–yellow (b∗) opponent colours. Therefore, the two last oppositions resemble the colour changes of the resasurin–resorufin indicators. However, the problem of quantitative compliance between these colour changes and the concentration/Uv-vis data is far from the complete practical resolution.

At the same time, there is an active recent interest in exploring the possibility of the usage of even more low-cost, mobile and accessible devices operating with a single-channel optical density only [38,39,40]. But in this case, establishing quantitative compliance with conventionalist spectrum-based data characterising colour-changing indicators is still an open problem.

**Figure 1:**
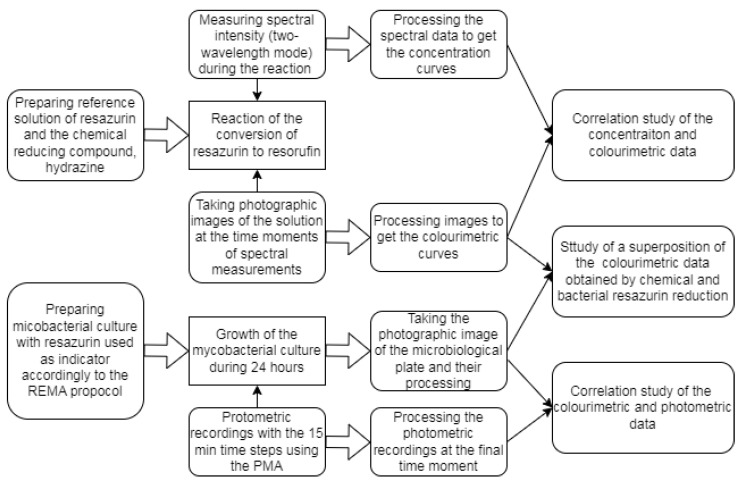
The flowchart of a sequence of investigations aimed at revealing correlation between UV-Vis-based, colourimetric and photometric data.

Thus, the present work addresses a sequence of such investigations as it is shown in Figure 1. As the first task, we will consider the sequential sets of spectrometric and colour photographic data corresponding to the process of the reduction of resazurin to resorufin by hydrazine hydrate, which is a colourless agent that provides a possibility to establish compliance between the indicators’ concentration changes and changes of intensity in the most appropriate colour space’s channel. During the second stage, we address a more applied problem using the data obtained with the recently developed portable microbiological analyser [41], which operates with the dynamic registration of the optical density in wells of a microbiological plate illuminated by a colour-filtered light source. Referring to the colourimetric conclusions of the first stage, we aim to explore the possibility of using such a simplified scheme for quantitative determinations of the minimal inhibitory concentration (MIC) by the case study of a drug-affected bacterial culture of *Mycobacterium tuberculosis*.

## 2. Materials and Methods

### 2.1. Compounds and Equipment

The basic solution of resazurin sodium salt was prepared as follows: (0.055±0.001)g of resazurin sodium salt (Sigma-Aldrich (Burlington, MA, USA), dye content >75%) was dissolved in distilled water, boiled and cooled to $\left(25\pm 2\right){\;}^{{}^{\circ}}C$. The mixture was stirred thoroughly until the substance was completely dissolved. The working solution of resazurin sodium salt was prepared by diluting the basic solution with boiled distilled water, thereafter cooled to the temperature $\left(25\pm 2\right){\;}^{{}^{\circ}}C$ in the ratio 1:2.5. The mass fraction of resazurin in the working solution was equal to 0.012%. Hydrazine hydrate (Acros Organics; hydrazine, 64%) was used as a reducing agent for resazurin. To increase the rate of the resazurin reduction reaction, hydrazine hydrate was taken with an excess of 100:1 in the molar ratio.

**Figure 2:**
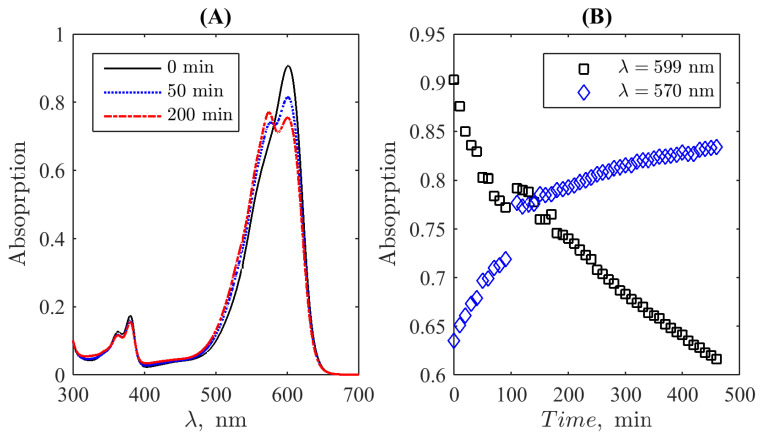
Examples of the evolution of the mixture’s spectrum during the transition from resazurin to resofufin induced by hydrazin (**A**) and the time course of spectral absorption intensity at the wavelengths 599nm and 570nm (**B**). The photometric uncertainty is equal to 0.004 absorption units in the used diapason, comparable to the size of markers.

To obtain the UV-Vis absorption curves, the Shimadzu UV-1800 (Shimadzu Corporation, Kyoto, Japan) spectrophotometer was used. The device is permanently routinely used in the Laboratory of Organic Synthesis at the Kursk State University and maintained in the calibrated state according to the technical specification; therefore, no special calibration was carried out. Distilled water was used as a reference solution. The mixture of resazurin and hydrazine hydrate was stirred continuously throughout the experiment to ensure uniform distribution of the dye and reducing agent concentration throughout the mixture. Figure 2A demonstrates typical examples of the spectrum’s evolution: the spectrum of the initial resazurin–hydrazine mixture (0 min), where the main peak is determined by resazurin, and two spectra recorded for some time moments left. They illustrate the development of the second principal peak due to the emergence of resorufin in the mixture due to the reaction between resazurin and hydrazine. Spectral absorption intensity at the wavelengths 599nm and 570nm was registered each at 10min in the spectrometer’s mode of recordings at two fixed lines, see Figure 2B. Simultaneously, the cuvette was photographed by an iPhone 13 camera for each round of two-line measurements. Pictures were taken in a chemical lab with the same “day-light”-type artificial ceiling illumination, the same for all time moments; the luminance balance of pictures respectively to the reference white background around the cuvette with a colour solution was controlled when the set of images for further processing was selected. The respective raw data are given in the Supplementary Materials.

For REMA experiments determining the minimal inhibitory concentration (MIC) of the standard first-line anti-tuberculosis drug isoniazid, the standard protocol of the broth microdilution in Middlebrook 7H9 as per the current guidelines of EUCAST [42] was applied to the reference strain *M. tuberculosis* H37Rv (ATCC 27294); the microplate was also photographed with the smartphone’s camera. The respective time-dependent photometric curves were obtained with a portable microbiological analyser (patent RU 2 779 840 C1), the construction of the principles of operation of which are described in the work [41].

### 2.2. Processing Absorption Spectra

The reference spectra of resazurin and resorufin were obtained from the AAT Bioquest database [43,44], respectively. Note that the absorption maxima correspond to about 599nm and 570nm for resazurin and resorufin, but both spectra are sufficiently wide. As a result, resazurin has 70% of the maximal absorption at the wavelength 570nm and resorufin 7% of the maximal absorption at the wavelength 599nm.

To take this into account, the registered values of absorption at these wavelengths $Y_{599}$ and $Y_{570}$ indicate not the direct concentrations of the compounds ($c_{Rz}$: reazurin; $c_{Rf}$: resorufin) but the linear combination stated in the standard way [45] as

(1) $$\begin{array}{cc} Y_{599} & =c_{Rz}+0.07c_{Rf}, \end{array}$$

(2) $$\begin{array}{cc} Y_{570} & =0.7c_{Rz}+c_{Rf}, \end{array}$$

where both spectra are normed to unity at their maxima.

Thus, the solution of the system (1) and (2) for each instant value of the registered spectral intensities gives the desired time course of the compounds’ concentrations:

(3) $$\begin{array}{cc} c_{Rz}\left(t\right) & =\frac{10}{951}\left(100Y_{599}\left(t\right)-7Y_{570}\left(t\right)\right), \end{array}$$

(4) $$\begin{array}{cc} c_{Rf}\left(t\right) & =\frac{10}{951}\left(100Y_{570}\left(t\right)-70Y_{599}\left(t\right)\right). \end{array}$$

### 2.3. Processing Colour Images and Photometric Data

The sequence of photographic pictures of the UV-Vis cell, in which the reaction of hydrazine hydrate with resazurin took place, was processed with the standard functions of MATLAB: they were read by imread as RGB matrices and converted to the L*a*b* colour space with rgb2lab.

**Figure 3:**
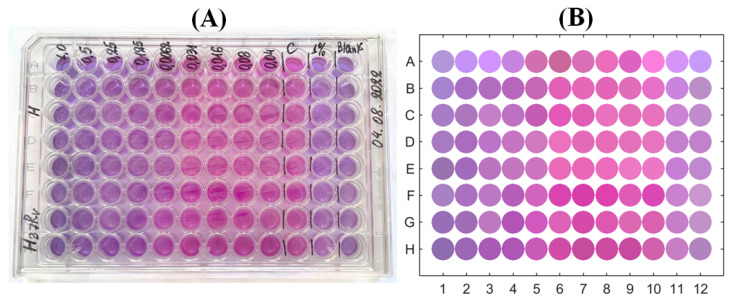
Photographic image of the microbiological plate after the end of the MIC-determining experiment (**A**) and its reconstruction by the colour extraction used for further quantification (**B**).

A similar procedure was applied for the colourimetric data obtained as a result of the experiment for determining the MIC. After its final stage, the microbiological plate was photographed (Figure 3A) and the obtained picture was processed with the ImageJ software. As the first stage, the general background-related illuminance correction was applied using the ’Process/Subtract Background’ procedure based on the rolling ball algorithm [46]. After this, the ’ReadPlate 3.0’ plugin [47] was used. It is specifically designed for the image processing of microbiological plates: the colour data were recorded by averaging over a circular region clearly inside each well’s boundary and taking into account the local blank correction, respectively, to the local wells’ surroundings, i.e., geometric and non-uniform illumination factors were eliminated. The results for a stack of colour channels of the RGB picture were converted to the CIE L*a*b* colour space with the separated colour channels, formed a table containing 96×3 array of data exported to a file, read by MATLAB and post-processed statistically. Figure 3B illustrates the picture of the microbiological plate obtained in this way.

The photometric data recorded by the portable microbiological analyser to a text file containing the time course of voltage from each light sensor were read by MATLAB and processed further with the home-made code, which uses standard functions of this software.

## 3. Results and Their Discussion

### 3.1. Compliance between UV-Vis and Colourimetric Data

**Figure 4:**
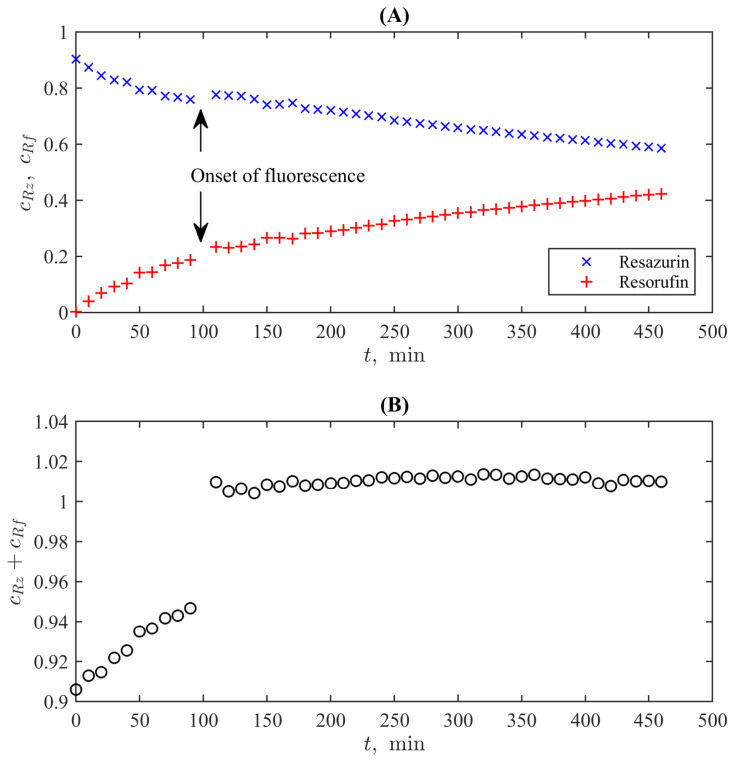
Time dynamics of the normed concentrations of resazurin and resorufin during the reaction of the former with hydrazine (**A**) and of the total concentrations of these compounds (**B**).

The resulting curves, which represent the normed concentrations of resazurin and resorufin calculated using Equations (3) and (4) from the UV-Vis spectrometric curves measured at lines 570nm and 599nm with the time course of the reaction (with the uniform time step of 10min), are shown in Figure 4A. They demonstrate the monotonous decay of resazurin’s concentration and the growth of that of resorufin. However, one can see an instant change from fast decay/growth to a slower (almost linear) one. An interesting observable fact is that the first stage of the process is not accompanied by resorufin’s fluorescence, and the change of the character of the dynamic curves occurs accurately at the onset of active fluorescence.

The absence of resorufin’s fluorescence by the excess amount of resazurin has been already reported in the literature [48,49] and especially in [28], where it is revealed that almost 90% of fluorescence is quenched when the ratio of resazurin to resorufin exceeds 20:1 in the course of an enzymatic reaction. In our experiment, this ratio is significantly lower than can originate from the usage of another redox agent, which also changes the initial pH of the solution. The latter unavoidably affects the colour change of the indicator and is traced by the UV-Vis analysis.

A complicated character of chemical processes during the first stage is confirmed by the plot shown in Figure 4B, which reports a sum of normed concentrations of two reagents, where the onset of fluorescence is expressed as a clear jump between the preceding and the following markers. It is valuable that the total concentration for t>110min is practically constant and deviates from unity by not more than 1%, which is within the expected experimental uncertainty of the UV-Vis absorption-based measurements. This is in complete accordance with the conventional kinetic model of resazurin reduction [50], which has the general form

(5) $$Rz+R\overset{k_{1}}{\to }Rf+RR\overset{k_{2}}{\underset{k_{2}^{-1}}{⇌}}DhRf,$$

where *R* and DhRf denote the redox reagent and dihydroresorufin, respectively.

Under conditions of a significant excess of the redox reagent’s concentrations, the direct reactions can be considered of the first order with the rate constants $K_{1}=k_{1}\left[R\right]$ and $K_{2}=k_{2}\left[R\right]$. In addition, in the considered system, the conversion from resorufin to dihydroresorufin (and its reverse counterpart) is significantly slower than the conversion from resazurin to resorufin and can be neglected during the time interval of the experiment. As a result, we obtain

(6) $$c_{Rz}=c_{Rz_{0}}\exp\left(-K_{1}t\right),\;c_{Rf}\approx c_{Rz_{0}}\left(1-\exp(-K_{1}t)\right),$$

i.e.,  $c_{Rz}+c_{Rf}=\mathrm{const}\equiv c_{Rz_{0}}$. The latter behaviour with the constant summary concentration is seen in Figure 4B for t>100min when the conventional conditions of the REMA test (with the fluorescence of resorufin) are fulfilled.

As for the first stage (t<100min), we can note two possible origins of the behaviour, which formally does not correspond to the basic reactions (5): (i) the fluorescence quenching may indicate more complex intermolecular interactions affecting the magnitudes of UV-Vis spectra for the studied wavelengths and (ii) the known fact that the elevation of pH leads to the changes of the spectra for both resazurin and resorufin in comparison to the condition of the standard spectral curves (pH÷6−7) [30]. Thus, an excess of resazurin can lead to the underestimation of concentrations via Equations (1) and (2) as it is seen in Figure 4. When the concentration of resorufin grows, these corrections compensate for each other. However, revealing the chemical background of this transient process is out of the direct scope of the present study, but simultaneously, its existence provides an additional test for establishing the compliance between UV-Vis and colourimetric approaches.

**Figure 5:**
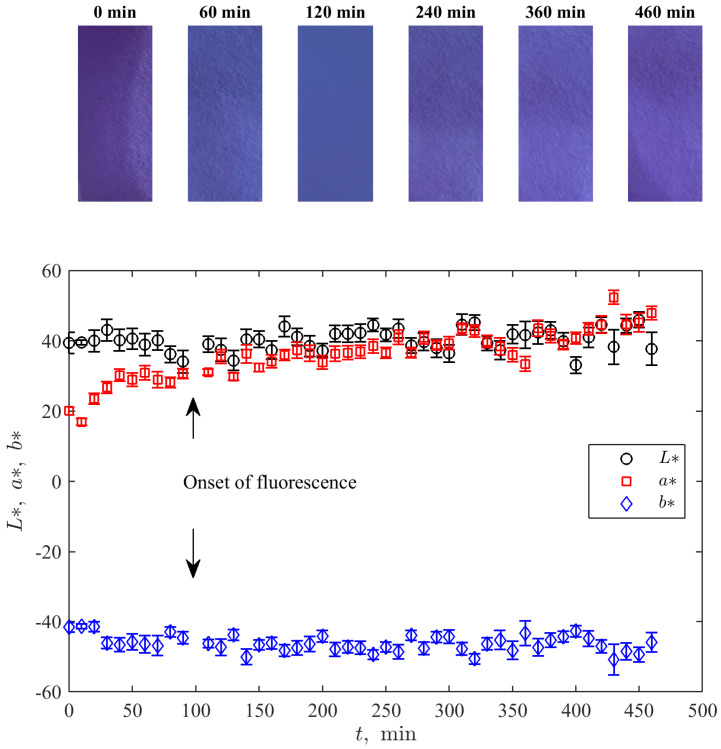
Time dynamics of the median values of the values corresponding to the channels of the CIE L*a*b* colour space for pictures of the experimental cell (several examples of such pictures for sequential time moments are shown over the plot). Error bars show the standard deviation.

Figure 5 demonstrates the time evolutions of the median intensity of channels in the CIE L*a*b* colour space [37] determined from photographic pictures of the cell (see some examples over the plot in Figure 5) taken at the same moment that the UV-Vis measurements were conducted. Here, L∗ measures the lightness, and a∗ and b∗ represent a degree of change from green to purplish red and from blue to yellow, respectively. Such a representation is especially suitable for the considered type of change of the indicator’s colour.

One can see an absence of a significant non-stationary trend for both L∗ and b∗ and a visible growth in the variable a∗. The latter corresponds to the growth of purplishness of the solution with the growth of resorufin’s amount. Moreover, the qualitative course of this sequence resembles the curve for resorufin’s concentration in Figure 4A: faster before the onset of fluorescence and more gradual thereafter.

**Figure 6:**
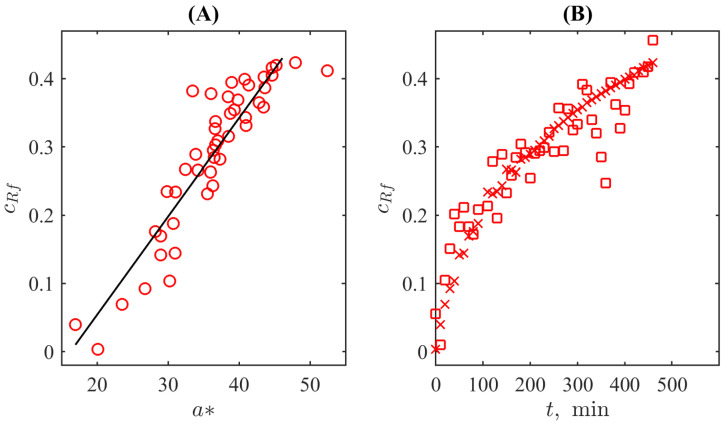
(**A**) The correlation plot between the values of a∗ colour channel and the normed concentration of resorufin. (**B**) The plot of resorufin’s concentration determined with the UV-Vis spectrometer (crosses corresponding to the same in Figure 4A) and by mapping a∗ values (squares corresponding to the same in Figure 5) to the concentration space (the linear map corresponds to the function shown in (**A**) as the straight line).

To quantify this resemblance, we plotted both dependences in one panel for the same time moments, see Figure 6A, where markers already follow a linear trend. The respective correlation coefficient is sufficiently high; it is equal to 0.91. The trend line is given by the equation $c_{Rz}=0.014a*-0.234$. The 95% confidence bounds are (0.012,0.016) for the slope and (−0.31,−0.16) for the shift, respectively. Taking into account that the colourimetric noise level is significantly higher than the UV-Vis spectrometer’s photometric accuracy, the detection limit is determined primarily by the former. By using the standard MATLAB function of uncertainty quantification predint, the detection limit corresponding to the defined parameters of the linear fit was determined as 0.1 of the relative concentration of resorufin in the solution at the level of standard uncertainty. Certainly, it is worse than can be achieved with the methods of spectral analysis but, in contrast to the problems of developing spectrometric biosensors aimed, in fact, at analytical chemistry in the strict sense (see, for example, [51]), this detection limit is accepted for conventional applications of the resazurin-based tests. For example, its principal usage (REMA) is the binary classification of the “growth/no growth” condition at the level either of 50% or 95% drug response [52]. Taking into account practical uncertainty connected with the reference microbiological population density in such tests, not only the first but also the second criterion can be satisfied with the obtained detection limit.

The obtained linear dependencedefines the mapping from the colourimetric representation to the UV-Vis spectrophotometry-based concentration as a function of the reaction time. Figure 6B illustrates this procedure: the raw median values of a∗ for each time moment are recalculated as UV-Vis-based concentrations and superposed with the results of the measurements. One can see that the resulting values represented by squares are uniformly scattered around the reference data represented by crosses (it represents the solution for resorufin’s concentration exponentially tending to saturation as follows from Equation (6)), especially in the region after the onset of fluorescence, i.e., in the conventional range of the REMA test. Before the onset of fluorescence, there is a certain (although not drastic) overestimation. It is in line with the feature, discussed above, that the UV-Vis-based spectra underestimate the concentration due to the specificity of the spectral response. On the contrary, the colourimetry detects “the degree of redness” directly originating from the presence of resorufin. Thus, colourimetry may be an even more relevant method for quantifying the process.

### 3.2. Compliance between Colourimetric and Optical Density Data

**Figure 7:**
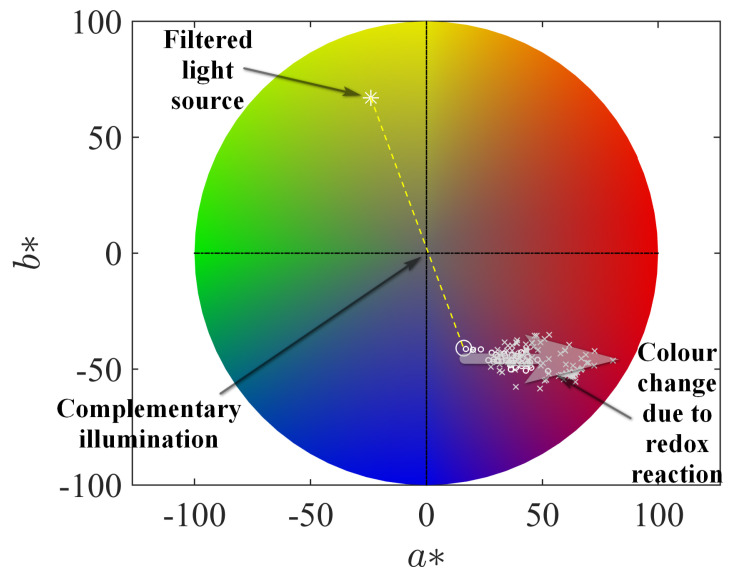
The colour wheel of the CIE L*a*b* scheme with the denoted position of the filter implemented in the portable microbiological analyser and colours of the resazurin–resorufin–hydrazine solution for different time moments, the same as in Figure 6 (positions marked by small circles) and of the microbiological plate’s cells (positions marked by small crosses) normed to the same intensity of the initial pure resazurin solution.

As the next step, we consider a more practical task of determining the minimal inhibitory concentration (MIC) for the action of isoniazid on the culture of *M. tuberculosis* with the application of a more simple optical procedure, which operates with the optical density under the specially adjusted colour-filtered illumination. In the portable microbiological analyser (PMA), the wells of the microplate were illuminated by yellow light due to the usage of a filter with the colour corresponding to the CIE L*a*b* triplet (96,−24,67); see Figure 7. In addition, this figure shows the positions of the point in the CIE L*a*b* colour space, which correspond to different concentrations of resazurin and resorufin in the solution (the luminance variable is adjusted to the value used for the PMA filter). One can see that the filter’s colour and resazurin’s solution colour are opposite in this colour space (a complementary illumination), i.e., the filtering procedure is adjusted to detect deviations originating from the linear shift from this initial state. This fact indicates that this simple and cheap practical realisation of filtering can be accepted for practical reasons of the PMA usage in medical centres not equipped with high-tech devices, as well as, say, for ecological studies in field conditions. Certainly, when it is possible, one can implement a more sophisticated realisation of better-adjusted colour filters, which are based, e.g., on nanostructured [53] or liquid crystal-based [54,55] filters with the controlled spectral characteristics.

**Figure 8:**
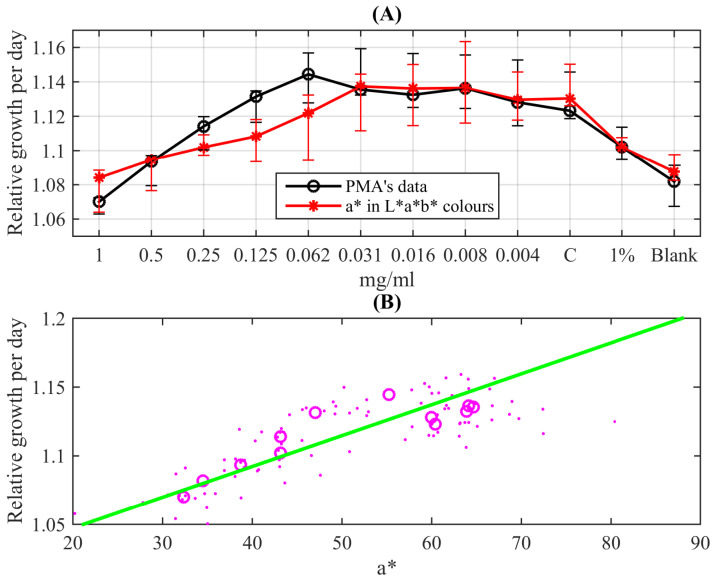
(**A**) REMA test curves obtained with the quantitative colourimetric (red asterisks) and the photometric (black circles) methods; the markers are connected by lines for the visual guidance. (**B**) The correlation plot between colourimetric data and the photometric relative growth (dots denote values for all 96 individual wells of the microbiological plate; circles denote median values for its rows.

Figure 8A demonstrates the obtained data characterising the response of the mycobacterial population to isoniazid in the standard form of the REMA test. They are obtained for this representation as follows: the voltage values from all 96 light sensors were recorded 24 h after resazurin was added to wells containing the suspension of *M. tuberculosis* and different dilutions of the drug (or control wells). These values were divided by the median of four voltage values recorded every 15 min during the first hour from the resazurin’s addition. The median value was taken to smooth possible transient effects after the process’ start; since *M. tuberculosis* is a slow-growing culture, there are no significant culture growth effects during such a short time interval. The black circles in Figure 8A show median values of the obtained relative elevation of sensors’ illuminance due to the colour change of the indicator for each column containing eight wells; whiskers indicate 0.05 and 0.95 quantiles of the data (extended uncertainty range). One can see a clear quantitative change in the response to the diminishing drugs’ concentration: circles follow almost linearly (slightly concaved up) till the concentration 0.062µg/mL. After this, the circles are scattered around a horizontal line in the range (0.031–0.004) µg/mL and the adjacent raw control (C). This indicates practically unperturbed bacterial growth. Thus, one can conclude that this data sequence reveals the MIC of isoniazid for the standard strain H37Rv as being between 0.062µg/mL and 0.031µg/mL. This range corresponds to the standard values known as (0.025–0.05) µg/mL and the mean value 0.031µg/mL [14].

A comparison of this PMA-based MIC with the visual picture-based determination (accordingly to the procedure described in the works [9,10]), which addresses colour change from blue to pink in the plate’s rows, see Figure 3, also confirms the MIC equal to 0.031µg/mL (it is especially clearly seen in Figure 3B, where the colour of wells are explicitly extracted). To make a quantitative comparison to the colour-based redox reaction response, which, as demonstrated above, can be quantitatively coordinated with the resazurin content in the solution, we applied the following procedure.

The photographic image of the microbiological plate was opened with ImageJ as an RGB picture and converted to the L*a*b* colour space employing the same software with decomposition in three images corresponding to intensities of these channels separately. For further processing, only the image of a* channel data was used. The values of the respective intensities in the central areas of each of the 96 cells, blank-corrected respectively to their surrounding, were extracted with the plugin PlateRead [47] installed to ImageJ; the mean values for the regions of interest—circles with a diameter of 15 pixels—were determined in the grayscale mode of the reader and exported. Figure 7 illustrates that the change of the a∗ colour channel follows the same horizontal stripe pattern that was found for the change induced by the reference chemical reduction of resazurin by hydrazine as seen from overlapping the respective markers. As discussed above, this change is coordinated with the UV-Vis-based change in the emerging resorufin concentration. Thus, we can refer to the results of colourimetry as a quantitative method.

Figure 8B depicts the correlation plot of these exporter values of the a∗ channel intensity and the monochromatic relative growth values determined by the PMA: dots are 96 well-by-well comparisons forming a stripe-like cloud following a linear trend, and circles denote pairwise comparisons of median values for each column. The correlation coefficient between these median values is equal to 0.87. The linear trend, shown as the green straight line, is given by the linear function Rel.growth=0.0017a∗+1.02. The 95% confidence bounds are (0.0011,0.0024) for the slope and (0.99,1.06) for the shift, respectively. The mapping of the a∗-channel data to the relative growth distribution given by this function is shown as red asterisks in Figure 8A and supplied with error bars indicating 0.05 and 0.95 quantiles of the data.

One can see that both datasets overlap with the extended uncertainty range; especially accurate quantitative coincidence is seen for the second half of the data. However, for the first half corresponding to concentrations larger than 0.031µg/mL, one can see more qualitative correspondence indicating increasing growth with diminishing the concentration of the added antibiotics. But what is most important from the practical point of view is that the quantitative change in the response pattern (growing vs. almost constant corresponding to the untreated control) coincides in the cases of both methods and results in the standard reference MIC of isoniazid acting on the H37Rv strain of *M. tuberculosis* equal to 0.031µg/mL.

## 4. Conclusions and Outlooks

Recently, the resazurin reduction test was endorsed by the World Health Organisation [56] in the list of recommended methods for microbiological culture and drug-susceptibility testing, especially within the context of screening patients at risk for multidrug-resistant tuberculosis. It is especially highlighted that colourimetric redox indicator methods can be applied specifically for settings with limited access to sophisticated laboratory infrastructure and technical expertise.

In this work, we explored a sequence of especially uncomplicated, cheap and accessible implementations quantified, respectively, to the UV-Vis spectrophotometric standard characterisation of the principal redox reaction, with the comparative summary given in Table 1.

It is revealed that in the range of conversion, which is typical for the resazurin reduction-based range of the indicator’s conversion, the spectral line-determined concentration of the reaction product, resorufin, is highly linearly correlated (more than 90% correlation coefficient) with the intensity values of the a∗ channel of the CIE L*a*b* colour space for the respective photographic pictures of the reaction cell. This result indicates better practical applicability of the colourimetric test in comparison with approaches based on alternative colour schemes, the interpretation of which requires sophisticated multichannel-based analysis [24,35,57] and even the usage of machine learning [58].

Although the colourimetric resolution in the sense of resorufin’s concentration detection limit is worse than that of spectral methods of analytical chemistry, it is completely compatible with the requirements of the resazurin microtiter assay (REMA) aimed at determining the minimal inhibitory concentration (MIC) of drugs acting on a bacterial culture since it operates with the binary (growth/no growth) classification of the response to the logarithmic serial dilution of an acting substance. It is demonstrated by the case study of the response of the standard strain H37Rv of *M. tuberculosis* to the serial dilutions of the first-line drug isoniazid. At the same time, the procedure of quantitative processing colourimetric data provides an opportunity not only to detect the indicator’s colour change but also to obtain the quantitative dose–response curve, determine its parameters, and quantify the uncertainty of target parameters. Therefore, the obtained results support an opportunity to quantify the process using even a smartphone camera as the registering device and relatively simple and free software for quantitative data processing. This makes it possible to extend the network of medical stations where such tests can be carried out for those that are not equipped with expensive and bulky commercial spectrophotometers. In addition, photographic registration can be easily applied in the case of ecology microbiological field studies or farm-based food quality control carried out far from equipped laboratories.

Moreover, the possibility to perform this quantification with single-number intensity results in the further simplification of the registration process by applying yellow-filtered illumination and very cheap semiconductor luxmeters. The latter scheme was tested with the recently developed portable microbiological analyzer [41] by the case study of the response of the standard strain H37Rv of *M. tuberculosis* to the serial dilutions of the first-line drug isoniazid. The obtained minimal inhibitory concentration values coincide with the known reference standard, and the response curve is coordinated with the colourimetric curve and, respectively, with the resorufin concentration, indicating the viability of treated bacteria. The latter provides certain outlooks for future research aimed at a more detailed assessment of the processes accompanying the REMA test. It was highlighted recently [6] that one needs to distinguish between the metabolic and population growth processes during the resazurin reduction reaction when dealing with a viable culture. An application of the simple photometric registration of data taken with a fine temporal resolution (not only the initial and the final states of colouration), which provides data that are well-coordinated with the more accurate methods, can provide an opportunity to study the functional time dependence of the colour–chemical growth curve and, respectively, to build more detailed biochemical models.

## Figures and Tables

**Table 1:** Comparative information on the investigated approaches.

Characteristics/Method	Spectrophotometry	Colourimetry	Photometry
Equipment registering signals	Commercial spectrophotometer	Smartphone camera	Portable microbiological analyser [41]
Cost	High	Low	Low *
Mobility	Low	Very high	High
Sensitivity and accuracy	Suitable to determine indicator’s concentration on the level of physical chemistry standards	Suitable to determine relative indicator’s concentration growth on the level of requirements for microbiological screening	Suitable to determine relative indicator’s concentration growth on the level of requirements for microbiological screening
Automation	Low to medium **	Low	High

* The components required required to assemble this device are described in Ref. [41], their total cost is comparable with the same of an average modern smartphone. ** Depends on the spectrophotometer’s model; the device used in this study requires manual start of measurements for each time moment.

## Data Availability

The set of raw data is provided in the Supplementary Materials; the discussed photographic images are given in the paper’s text.

## Supplementary Materials

The following supporting information can be downloaded at https://www.mdpi.com/article/10.3390/bioengineering10080962/s1. The raw data were obtained by UV-Vis spectrometer in two-wavelength mode of recordings for 570nm and 599nm as well as images of the cuvette (UV_vis_and_photo.zip), and the set of photometric recordings by the PMA (each file corresponds to one of microplate’s well where the first column contains time moments separated by 15 min interval and the second column contains the raw voltage recorded by a luxmeter (PMA_data.zip).
